# Stability of Respiratory Syncytial Virus in Nasal Aspirate From Patients Infected With RSV

**DOI:** 10.1111/irv.70058

**Published:** 2024-12-16

**Authors:** Atsuko Yamamoto, Yoko Hayasaki‐Kajiwara, Takamichi Baba, Saori Okaga, Mayumi Kakui, Takao Shishido

**Affiliations:** ^1^ Laboratory for Drug Discovery and Disease Research Shionogi & Co, Ltd Osaka Japan; ^2^ Biostatistics Center, Shionogi & Co, Ltd Osaka Japan; ^3^ Shionogi TechnoAdvance Research & Co, Ltd Osaka Japan

**Keywords:** nasal aspirates, respiratory syncytial virus, virus stability, virus titer

## Abstract

**Background:**

Evaluation of infectious virus titer is a challenge for respiratory syncytial virus (RSV) clinical trials because of the labile nature of RSV and rapid loss of infectivity without proper specimen handling. However, there has been no rigorous investigation into RSV stability in clinical specimens.

**Methods:**

RSV stability was investigated by evaluating virus titers and defined as titer variation from baseline within three standard deviations of our titration assay. RSV stability in viral transport medium (VTM) at 4°C and the effect of freezing method on stability were evaluated using RSV‐A2 stock. RSV stability in nasal aspirates collected in VTM at 4°C was estimated by regression analysis of virus titers measured at several time points. Stability of these specimens stored at −80°C for 10–15 months after freezing by the method, which maintained RSV‐A2 stability, was also assessed.

**Results:**

Three standard deviations were calculated from our titration assay as 0.97 log_10_ 50% tissue culture infectious dose (TCID_50_/mL), and RSV stability was defined as variation of virus titer from baseline within 1.0 log_10_TCID_50_/mL. RSV‐A2 in VTM at 4°C was stable for at least 120 h. Freezing at −80°C negatively affected virus stability, whereas freezing in liquid nitrogen or a dry ice‐ethanol bath did not. RSV in nasal aspirates was stable for 2 days at 4°C and for 10–15 months at −80°C after snap freezing.

**Conclusions:**

RSV in nasal aspirates in VTM was estimated to be stable for 2 days at 4°C and for approximately 1 year at −80°C.

## 1 Introduction

Respiratory syncytial virus (RSV) is the leading cause of serious acute lower respiratory infection (ALRI) in infants. The World Health Organization (WHO) estimates the global burden of RSV‐associated ALRIs to be at 33 million annually, resulting in more than 3 million hospitalizations and 26,330 RSV‐associated ALRI in‐hospital deaths in children aged 0–60 months. Especially infants aged 0–6 months, RSV‐associated ALRI accounts for approximately 1.4 million hospitalizations and 13,300 in‐hospital deaths [1, 2]. Currently, there are three US Food and Drug Administration‐approved vaccines for adults: Abrysvo, Arexvy, and mRESVIA [3, 4, 5] and, for infants, palivizumab and nirsevimab, which are monoclonal antibodies [6, 7], and maternal vaccine Abrysvo [3]. However, the target populations of these newly approved vaccines and antibodies are limited [8]. In addition, current treatment options are limited to ribavirin, which provides questionable benefit [9] and supportive care. Therefore, there is a great need to develop novel therapeutic agents against RSV. Furthermore, because it is well known that children are often exposed to and infected with RSV outside the home, such as in school or childcare centers [10], and RSV is rapidly transmitted within these childcare facilities [11], antiviral agents against RSV are expected to not only alleviate the symptoms of patients but also to reduce RSV transmission from patients to others.

Infectious virus titer is an important virological parameter for the investigation of antiviral efficacy of therapeutic agents and has been evaluated in several clinical trials for respiratory viruses, such as influenza and SARS‐CoV2 [12, 13]. In particular, because virus infectivity is one of the factors that determine transmissibility [14], infectious virus titer has been evaluated to investigate the efficacy of therapeutic agents at preventing transmission of respiratory viruses [15, 16, 17]. However, evaluation of infectious RSV titer is a challenge in clinical trials because RSV is known to be a labile virus and rapidly loses viral infectivity unless specimens are properly handled [18, 19]. WHO recommends RSV specimens to be immediately stored in viral transport medium at 4°C. If specimens cannot be processed within 48 h, it is recommended that they are stored at or below −70°C [20]. However, stability of RSV in clinical specimens stored under these conditions has not been rigorously investigated. In this study, we therefore investigated the stability of virus in nasal aspirates collected from patients and stored in viral transport medium (VTM) at 4°C or frozen by evaluating infectious virus titer.

## 2 Methods

### 2.1 Cell Culture and RSV‐A2 Stock Preparation

HEp‐2 cells (American Type Culture Collection [ATCC]) were cultured in minimum essential medium (MEM) containing 10% fetal bovine serum (FBS) and 1% kanamycin. RSV‐A2 (VR‐1540, ATCC) was propagated in HEp‐2 cells cultured in MEM containing 2% FBS and 1% kanamycin (viral assay medium). After the propagation, the virus was harvested with viral assay medium. After that, 40% sucrose in PBS was added (final concentration of sucrose was 10%) and aliquoted to yield RSV‐A2 stocks. These RSV‐A2 stocks were snap frozen in a dry ice‐ethanol bath and stored at −80°C until use.

### 2.2 Measurement of Virus Titer

One day prior to virus infection, HEp‐2 cells were seeded on 96 well plates in viral assay medium. The samples to be titrated were diluted with the viral assay medium with tenfold serial dilution scheme. The cells were then inoculated with the diluted samples and incubated at 37°C in 5% CO_2_ for 2 h. Cells were then washed, and 100 μL per well of viral assay medium was added and incubated at 37°C in 5% CO_2_ for 3 days. Cells were then fixed in 4% paraformaldehyde phosphate buffer solution, followed by incubation in blocking buffer for 1 h at room temperature. The blocking buffer was then removed, and a 1000‐fold dilution of primary goat anti‐RSV antibody (Bio‐Rad Laboratories) was added. After 1‐h incubation at room temperature, the cells were washed with Dulbecco's phosphate buffered saline (DPBS), and a 2000‐fold dilution of horseradish peroxidase‐conjugated secondary goat IgG antibody (R&D Systems) was added. After the incubation for 1 h at room temperature, the cells were washed with DPBS, and 1 Step Ultra TMB‐Blotting solution (Thermo Fisher Scientific) was added. After incubation at room temperature for 15 min, the cells were washed with water. The presence of stained virus‐infected cells was observed under a stereo microscope. Virus titer was calculated as 50% tissue culture infectious dose (TCID_50_)/mL using the Reed‐Muench method [21].

In each virus titration assay of clinical specimens, aliquoted RSV‐A2 stock was also titrated as an assay control.

### 2.3 Measurement of Virus RNA

Virus RNA was extracted from samples using a Quick RNA viral Kit (ZYMO RESEARCH) in accordance with the manufacturer's protocol. RSV RNA copy number was measured by quantitative RT‐PCR using the QuantiTect SYBR Green RT‐PCR kit (QIAGEN) and qPCR primer pairs targeting the N gene [22] (forward: 5′‐CATCCAGCAAATACACCATCCA‐3′, reverse: 5′‐TTCTGCACATCATAATTAGGAGTATCAA‐3′).

### 2.4 Assessment of RSV Stability Using RSV‐A2 Laboratory Strain

To assess the stability of RSV‐A2 in VTM at 4°C, the RSV‐A2 stock was diluted 100 or 10,000‐fold in viral assay medium. The virus stock dilution was added to VTM (Universal Viral Transport, Becton, Dickinson and Company) at a volume ratio of 1:100 at 120, 96, 72, and 48 h prior to virus titration and stored at 4°C. These samples were titrated in parallel with samples prepared just before virus titration. The impact of freezing method on virus stability was evaluated using a 100‐fold dilution of RSV‐A2 stock added to VTM. Six‐hundred microliter samples were then aliquoted into 2‐mL tubes and frozen in a −80°C deep freezer, in liquid nitrogen, or in a dry ice‐ethanol bath followed by virus titration.

### 2.5 Assessment of RSV Stability in Nasal Aspirates Collected From Patients Infected With RSV

Nasal aspirates were collected from 16 patients under 5‐years of age who had respiratory symptoms and confirmed RSV infection by a rapid diagnostic test at the Kids clinic YAMAMOTO in Osaka during the 2019/2020 season.

After the collection of nasal aspirates from each patient, a sterile cotton swab was dipped into the nasal aspirate and placed into VTM. These clinical specimens were stored at 4°C at the clinic until they were transported to our laboratory by refrigerated shipping. After the clinical specimens arrived at our laboratory, fresh VTM at 4°C was added, and 300 and 600 μL of each specimen were then aliquoted into 2‐mL tubes. The aliquots for the assessment of virus stability at 4°C or virus stability after snap freezing were stored at 4°C until use. The remaining 600 μL per tube aliquots were snap frozen in liquid nitrogen and stored at −80°C.

For assessment of virus stability in clinical specimens stored at 4°C, 300 μL per tube aliquots were titrated on the day they arrived at our laboratory. The other 300 μL per tube aliquots stored at 4°C were titrated at an additional one or two time points within 9 days of sample collection.

To evaluate the impact of snap freezing on virus stability, 600 μL per tube aliquots stored at 4°C were snap frozen in liquid nitrogen. After snap freezing, these aliquots were titrated in parallel with other 300 μL per tube aliquots stored at 4°C without freezing. Virus stability after long‐term storage at −80°C was evaluated using the aliquots snap frozen in liquid nitrogen on the day they arrived at our laboratory and stored at −80°C.

### 2.6 Statistical Analysis of Virus Titer of Clinical Specimens With a Mixed Effect Regression Model

The virus titer of clinical specimens was statistically analyzed using a mixed effect regression model. To model the relationship between RSV titer of a clinical specimen stored at 4°C and storage period, the regression model shown below was conducted (see the Supporting Information regarding adoption of the model):

$$y_{i,\mathrm{day}}=\alpha +a_{i}+\left(\beta +b_{i}\right)*\mathrm{day}+\varepsilon_{i},$$

where i is the sample number and $\mathrm{day}\in \left[1,10\right]$ is the storage period. α and β are the fixed effects of intercept and slope, respectively. $a_{i}$ and $b_{i}$ are the random effects on intercept and slope specific to the sample, respectively. $\varepsilon_{i}$ is the error term. Let ($a_{i}$, $b_{i}$) and $\varepsilon_{i}$ follow a normal distribution with mean 0 and let the unstructured variance–covariance matrices of the simultaneous distributions of $a_{i}$ and $b_{i}$ be considered. $\left(a_{i},b_{i}\right)$ and $\varepsilon_{i}$ were assumed to be independent.

Considering the variability of the data, the period during which the lower limit of the two‐sided 90% confidence interval of the predicted mean virus titer did not decrease from the predicted viral titer on Day 1 beyond the reference value set for virus stability was assessed. This period was predicted as the duration when RSV in clinical specimen was stable.

## 3 Results

### 3.1 Variability of Virus Titration Assay Data and Definition of RSV Stability

To assess the variability of data from our virus titration assay, three RSV‐A2 stocks were titrated in each three independent experiments, and standard deviation (SD) was calculated (Table 1, SD = 0.323). Based on the three‐standard‐deviation value as 0.97 log_10_TCID_50_/mL, RSV was defined to be stable when the variation of virus titer from baseline was within 1.0 log_10_TCID_50_/mL.

**TABLE 1:** Variability of virus titration assay data.

	Virus titer of RSV‐A2 stocks (log_10_TCID_50_/mL)			
	Exp. 1	Exp. 2	Exp. 3	Mean ± SD
Stock 1	7.8	7.3	7.8	7.9 ± 0.3
Stock 2	8.1	8.5	7.6	
Stock 3	8.1	7.8	7.8	

*Note:* Three RSV‐A2 stocks were titrated in each independent experiment and the mean ± SD was calculated. Nine RSV‐A2 stocks were titrated in total.

Abbreviation: Exp., experiment.

### 3.2 Assessment of RSV Stability Using RSV‐A2 Laboratory Strain

**FIGURE 1:**
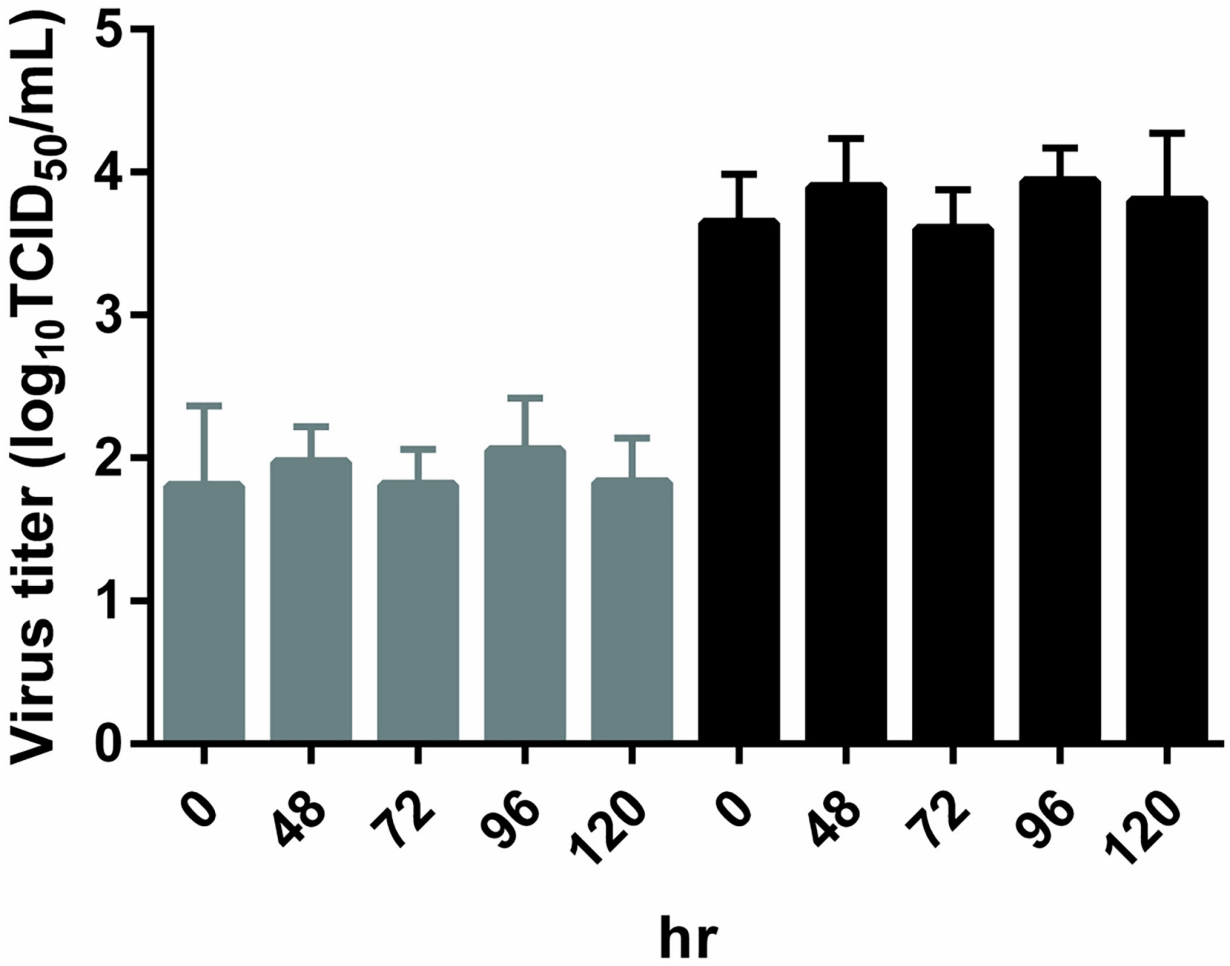
Virus titer of RSV‐A2 added to VTM and stored at 4°C. RSV‐A2 stock dilutions of 100‐ or 10,000‐fold were added to VTM and stored at 4°C for 0, 48, 72, 96, and 120 h. Infectious virus titers were then measured. Four samples were titrated at each time point, and data are presented as the mean ± SD. Gray bars: samples containing 10,000‐fold diluted RSV‐A2 stock. Black bars: samples containing 100‐fold diluted RSV‐A2 stock. Baseline: virus titer of the samples prepared just before virus titration (0 h).

**FIGURE 2:**
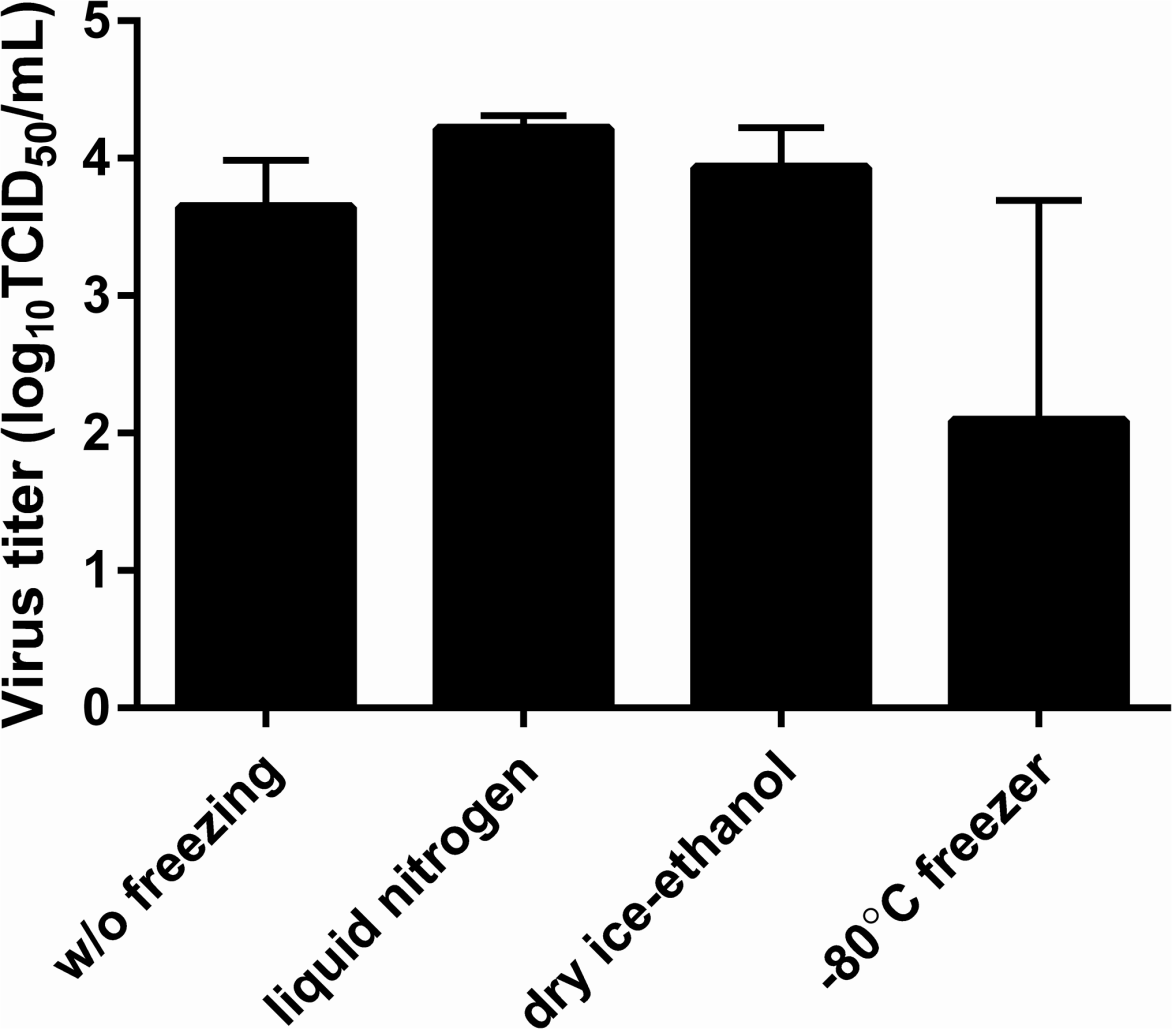
Virus titer of RSV‐A2 added to VTM and frozen in liquid nitrogen, in a dry ice‐ethanol bath, or in a −80°C freezer. RSV‐A2 stock diluted 100‐fold was added to VTM and frozen in liquid nitrogen, in a dry ice‐ethanol bath, or in a −80°C freezer for approximately 3 h. The infectious virus titer of these samples was then measured. Three samples were titrated for each freezing condition, and four samples were titrated for the condition without freezing. Data are represented as the mean ± SD. Baseline: virus titer of the samples without freezing (same data as the black bar at 0 h in Figure 1).

Prior to the investigation of RSV stability in nasal aspirates, stability of RSV‐A2 laboratory strain in VTM at 4°C was evaluated (Figure 1). For 120 h, virus titers of the samples containing 100 or 10,000‐fold dilutions of RSV‐A2 stock varied within 1.0 log_10_TCID_50_/mL from those of the samples prepared just before virus titration. This result indicated that RSV‐A2 propagated in HEp‐2 cells was stable for at least 120 h in VTM at 4°C. The effect of freezing method on virus stability of these RSV‐A2 samples was evaluated by freezing the samples either in a −80°C freezer for approximately 3 h, in liquid nitrogen, or in a dry ice‐ethanol bath (Figure 2). Virus titer of the samples frozen in a −80°C freezer was approximately 2.0 log_10_TCID_50_/mL lower than that of the samples without freezing, whereas the amount of virus RNA was stable (see Figure S1). The virus titer of the samples frozen in liquid nitrogen or in a dry ice‐ethanol bath did not change more than 1.0 log_10_TCID_50_/mL from that of the samples prepared just before virus titration. These results indicate that slow freezing in a −80°C freezer affected RSV stability whereas snap freezing in liquid nitrogen or dry ice‐ethanol bath maintained RSV stability.

### 3.3 RSV Stability in Nasal Aspirate Collected From Patients Infected With RSV

**FIGURE 3:**
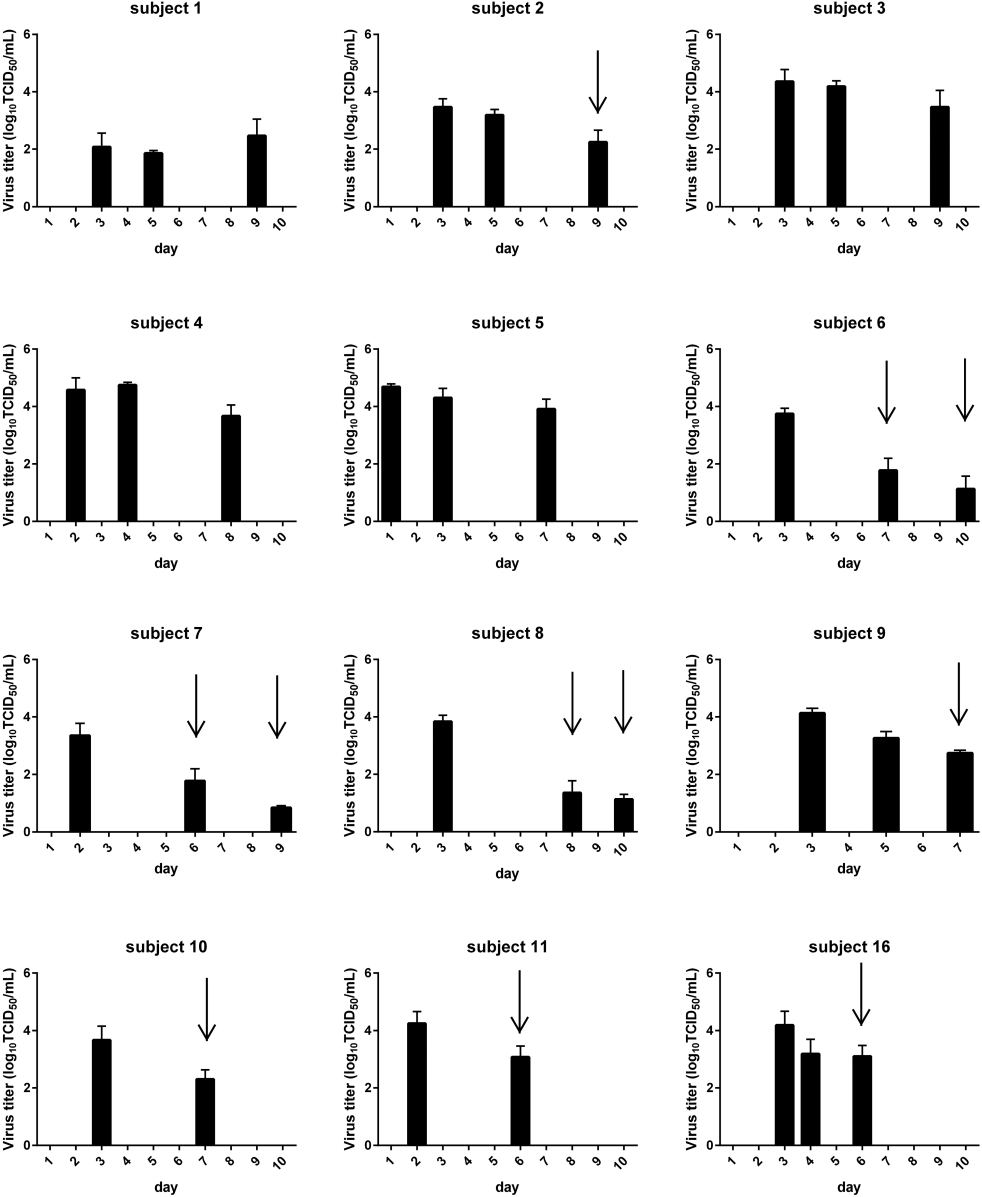
Virus titers of nasal aspirates from each subject collected in VTM and stored at 4°C. Nasal aspirates from patients infected with RSV were collected in VTM and stored at 4°C. Virus titers of these specimens were measured at two or three time points within 9 days from the day of nasal aspirate collection. Three aliquots of each specimen were titrated at each time point, and the data are represented as the mean ± SD. Baseline: virus titer of each subject at first time point. Black arrow: Virus titer was more than 1.0 log_10_TCID_50_/mL lower than the baseline. See the Supporting Information regarding Subjects 12–15.

The stability of RSV in nasal aspirates collected from patients with RSV and stored in VTM at 4°C was assessed by titrating these clinical specimens at two or three time points within 9 days from the day of collection (Figure 3). From 3 days after the first measurement, virus titers of some specimens were more than 1.0 log_10_TCID_50_/mL lower than that at the first measurement. These results indicated that RSV in nasal aspirates stored in VTM at 4°C was stable for 2 days.

**FIGURE 4:**
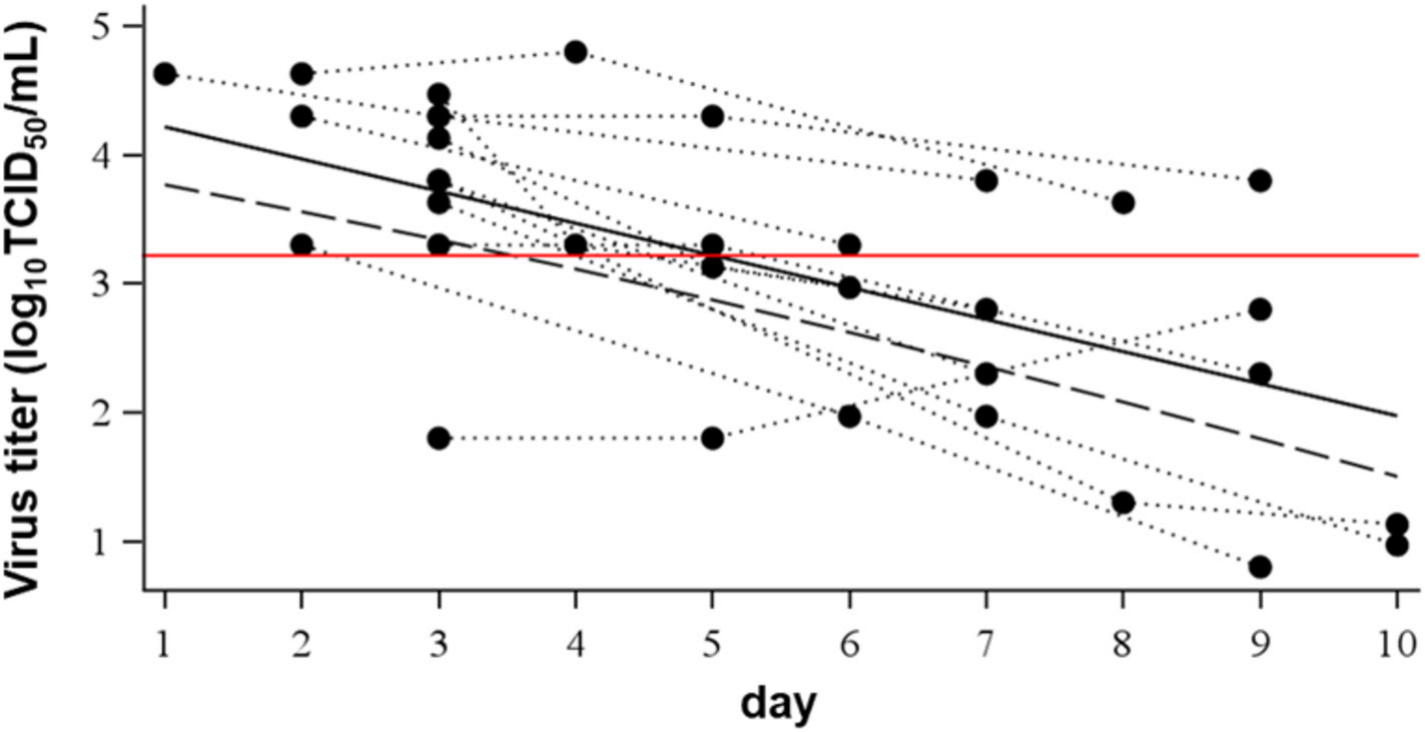
Mixed effect regression model‐based predicted virus titer of clinical specimens. Solid black and red lines: regression model‐based predicted mean virus titer and its lower limit of two‐sided 90% confidence interval, respectively (same data as model‐based values in Table 2). Dashed‐dotted line: the model‐based predicted virus titer on Day 1 minus 1.0 log_10_TCID_50_/mL. Each plot shows the observed virus titer of each subject (same data as that in Figure 3).

**FIGURE 5:**
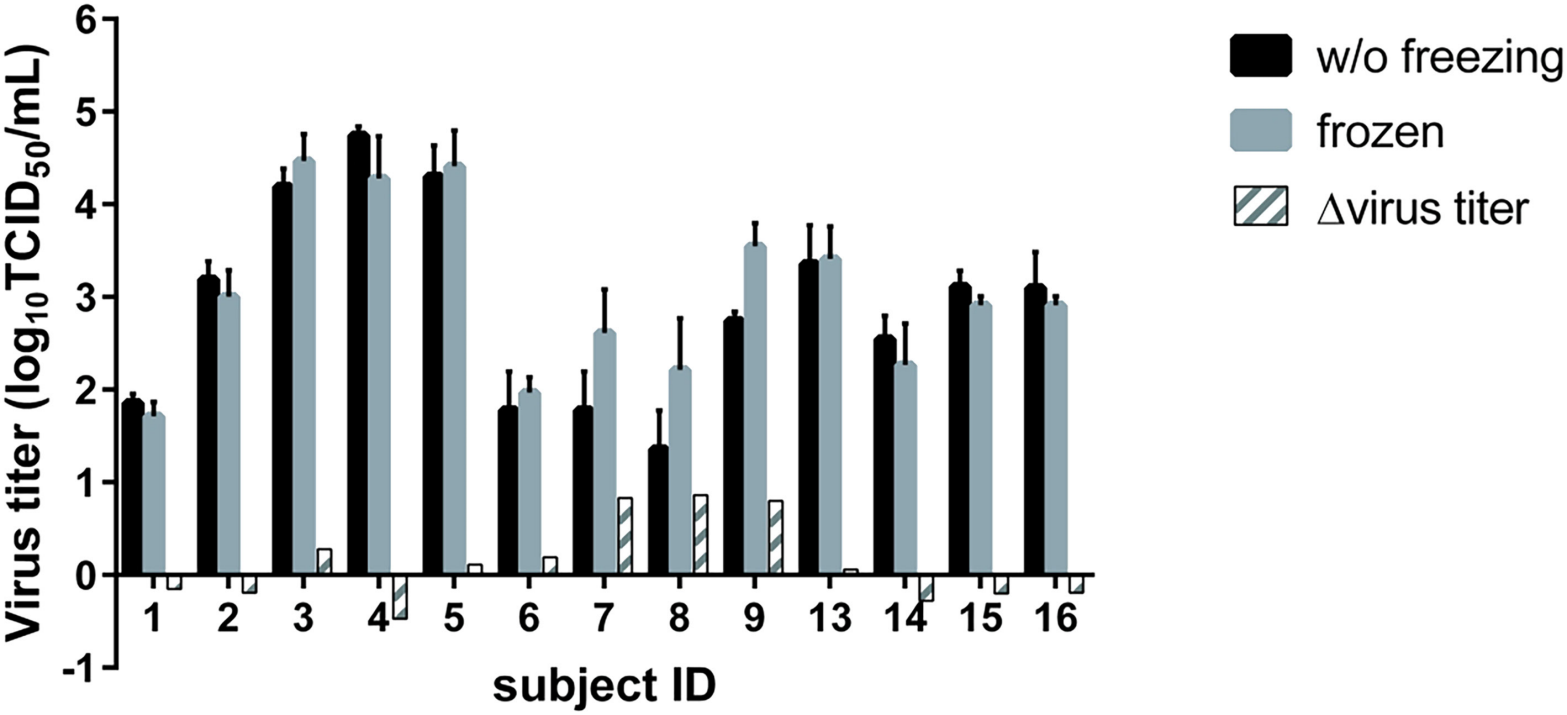
Virus titer of clinical specimen from each subject stored with or without freezing in liquid nitrogen. Aliquots of clinical specimen from each subject were snap frozen in liquid nitrogen and thawed on the same day. After thawed, these aliquots were titrated in parallel with other aliquots stored without freezing. Three aliquots were titrated for each subject, and data are represented as the mean ± SD. Baseline: virus titer of the samples without freezing for each subject. Assessment was not conducted for Subjects 10–12.

The duration of RSV stability in specimens stored at 4°C was estimated by analyzing these observed virus titration data using a regression model. The mean virus titer on each day and their lower limit of two‐sided 90% confidence intervals predicted by this model are shown in Table 2 and Figure 4. The lower limit of the two‐sided 90% confidence interval on Day 4 first decreased more than 1.0 log_10_TCID_50_/mL than the model‐based predicted virus titer on Day 1, indicating that RSV in clinical specimens stored at 4°C was stable for 2 days. Overall, both the observed virus titer of each specimen and the model‐based analysis indicated that RSV in nasal aspirate collected and stored in VTM at 4°C was stable for 2 days. The effect of snap freezing on the stability of RSV in these clinical specimens was investigated by freezing clinical specimen aliquots in liquid nitrogen (Figure 5). None of the snap frozen specimens showed more than a 1.00 log_10_TCID_50_/mL reduction in virus titer compared with samples without freezing, indicating that snap freezing in liquid nitrogen did not affect the stability of RSV in nasal aspirate.

**TABLE 2:** Observed and mixed‐effect regression model–based predicted mean values of clinical specimen virus titers on each day.

Day	Observed value		Model based value	
	*n*	Mean (SD)	LS mean (SE)	[90% confidence interval]
1	1	4.63 (—)	4.21 (0.28)	[3.71, 4.71]
2	3	4.08 (0.69)	3.97 (0.25)	[3.53, 4.41]
3	9	3.73 (0.81)	3.72 (0.22)	[3.33, 4.12]
5	4	3.13 (1.03)	3.24 (0.20)	[2.88, 3.60]
6	3	2.75 (0.69)	3.00 (0.21)	[2.63, 3.37]
7	4	2.72 (0.80)	2.76 (0.22)	[2.35, 3.16]
8	2	2.47 (1.65)	2.51 (0.25)	[2.06, 2.97]
9	4	2.43 (1.25)	2.27 (0.29)	[1.76, 2.78]
10	2	1.05 (0.11)	2.03 (0.32)	[1.45, 2.61]

*Note:* Model‐based values were plotted in Figure 4.

Abbreviation: SE, standard error.

RSV stability in these clinical specimens after long‐term storage at −80°C was also assessed by titrating the clinical specimen aliquots which were snap frozen and stored at −80°C for 10–15 months (Table 3). No specimen showed more than a 1.00 log_10_TCID_50_/mL reduction in virus titer from that measured on the day they arrived at our laboratory. This result indicated that RSV in nasal aspirate collected in VTM was stable after snap freezing and storage at −80°C for 10–15 months.

**TABLE 3:** Virus titers of clinical specimens on the day of arrival at our laboratory and after storage at −80°C for 10–15 months.

Subject ID	1	2	3	4	16
Virus titer on the day of arrival at our laboratory (log_10_TCID_50_/mL)	2.08 ± 0.48	3.47 ± 0.29	4.36 ± 0.42	4.58 ± 0.42	4.19 ± 0.48
Virus titer after storage at −80°C (log_10_TCID_50_/mL)	1.63	3.30	4.80	4.63	3.80
Storage period (months)	15	15	15	15	10

*Note:* Baseline: virus titer measured on the day of sample arrival at our laboratory. Three aliquots were titrated on the day of sample arrival at our laboratory, and the data are represented as the mean ± SD. One aliquot was titrated after storage at −80°C.

## 4 Discussion

In this study, the stability of RSV in nasal aspirates collected from patients with RSV and stored in VTM was investigated by evaluating infectious virus titer. Our investigation indicated that RSV in nasal aspirate collected in VTM was stable for 2 days when stored at 4°C and stable for approximately 1 year when stored at −80°C after snap freezing.

Recently, Kitai et al. [23] reported that the RSV isolation rate from clinical specimens, such as nasal swabs, nasal aspirates, and throat swabs, stored in transport medium at 4°C began to decrease from the day following sample collection indicating that RSV is stable in clinical specimens for less than 2 days. One potential reason for the shorter duration of stable RSV in the study of Kitai et al. compared with our study is the difference in the components of the transport medium. Kitai et al. used MEM containing 0.5% gelatin, which has the potential to stabilize RSV [24], as VTM. The VTM used in this study contained not only gelatin but also sucrose and bovine serum albumin (BSA) [25]. Sucrose and FBS, the major component of which is BSA, provide better stabilization to RSV than gelatin [24]. Therefore, we consider that these VTM components led to the longer stability of RSV in this study.

Prior to the investigation of RSV stability in nasal aspirates, the stability of RSV‐A2 laboratory strain stored in VTM at 4°C was investigated and it was shown that RSV‐A2 was stable for at least 120 h. In the study of van der Gucht et al. [26], stability of some RSV isolates stored at 4°C was shorter than that in our study. In their study, DMEM without FBS was used as VTM. As discussed regarding the difference of the clinical specimen stability between our study and Kitai's study, we consider the difference of VTM components between two studies possibly affected the results. However, head‐to‐head comparison of RSV stability in the VTM used in each study is considered to be beneficial for better understanding of the effect of VTM components on RSV stability.

In this study, although the virus titers of RSV‐A2 samples were almost the same as those of clinical specimens, RSV‐A2 was stable for a longer period than RSV in nasal aspirates. It is not clear why RSV‐A2 was stable than RSV in nasal aspirates, but it is possible that some components in the nasal aspirate affected virus stability. For example, mucus production is increased in patients infected with RSV [27]. Therefore, nasal aspirates from patients with RSV are considered to contain mucus. In contrast, stocks of RSV‐A2 propagated in HEp‐2 cells do not contain mucus because HEp‐2 cells are not mucus producing cells, unlike goblet cells and mucus cells [28].

Mucus in nasal aspirate from patients infected with RSV contains mucins as a major structural component and several microbicidal enzymes, such as elastase, proteinase‐3, cathepsin G, lysozyme, and myeloperoxidase [29, 30, 31, 32]. Among these components, elastase, proteinase‐3, and cathepsin G inhibit RSV infection of HEp‐2 cells. In particular, elastase and proteinase‐3 cleave F protein of RSV, which plays a pivotal role in virus infection [32]. Moreover, mucins, which are highly glycosylated proteins, can affect virus stability because they directly interact with viruses, such as influenza viruses and coronaviruses, via mucin glycans and inhibit their ability to infect epithelial cells [33, 34]. However, it must be taken into consideration that the clinical specimens in this study were prepared by adding nasal aspirate to VTM; thus, the concentration of these components was much lower than in the nasal aspirate itself. In addition, the activity of these enzymatic components at 4°C is unknown.

Our investigation into the effect of freezing method on virus stability showed that slow freezing of the samples in a −80°C freezer negatively affected RSV stability, whereas snap freezing using liquid nitrogen or a dry ice‐ethanol bath maintained RSV stability. During slow freezing, protein denaturation is caused by solutes in the unfrozen water phase concentrating more gradually and causing larger ice crystals than during snap freezing [35]. Therefore, RSV surface proteins, such as F protein and G protein, which mediate virus attachment and entry into cells [19], were probably denatured during slow freezing in a −80°C freezer.

We investigated the stability of RSV in nasal aspirates using specimens collected during the 2019/2020 season. Our samples consisted mainly of Subtype A except one specimen of subtype B (Subject 1: Subtype B; all other subjects: Subtype A). Therefore, a limitation of our study is that we did not investigate whether the RSV stability in clinical specimens is affected by the season and/or specific characteristics of viral subtypes. Despite this limitation, the investigation of RSV stability in clinical specimens stored at 4°C or −80°C, which are feasible storage conditions in a clinical setting, is helpful for understanding proper handling of clinical specimens to maintain virus stability. Furthermore, our findings will contribute the accurate evaluation of antiviral therapeutic agents and better understanding of the potential of the agents that prevent virus transmission.

## Author Contributions

**Atsuko Yamamoto:** methodology, formal analysis, investigation, resources, validation, visualization, writing – original draft. **Yoko Hayasaki‐Kajiwara:** project administration, methodology, writing – review and editing, investigation, resources. **Takamichi Baba:** formal analysis, methodology, validation, visualization, writing – original draft. **Saori Okaga:** formal analysis, investigation, writing – review and editing. **Mayumi Kakui:** investigation, writing – review and editing, formal analysis. **Takao Shishido:** conceptualization, supervision, writing – review and editing.

## Ethics Statement

This study followed the tenets of the Declaration of Helsinki and the Governmental Guidelines and was approved by the Institutional Human Research Ethics Committee.

## Consent

Informed consent has been obtained from the legal representative of the patient for use of the specimens in this study.

## Conflicts of Interest

All authors are employees of Shionogi & Co., Ltd., or its subsidiary company Shionogi TechnoAdvance Research Co., Ltd.

### Peer Review

The peer review history for this article is available at https://www.webofscience.com/api/gateway/wos/peer‐review/10.1111/irv.70058.

## Supporting information


**Figure S1** RNA of RSV‐A2 Laboratory Strain added to VTM and Frozen in a − 80 °C freezer. One‐hundred‐fold dilution of virus stock was added to VTM and frozen in a − 80 °C freezer (*n* = 4). Virus RNA was then measured and is expressed as the mean ± SD. Baseline:virus RNA of samples prepared just before RNA measurement [without (w/o) freezing, *n* = 2; data is expressed as the mean].

**Figure S2** Virus Titers of Nasal Aspirates from Subjects 13, 14 and 15 Collected into VTM and Stored at 4 °C. Three specimen aliquots were titrated at each time point and data are represented as the mean ± SD. Baseline:virus titer at first measurement time point of each subject. Black arrow:the sample showed more than 1.0 log_10_TCID_50_/mL lower virus titer from the baseline.

**Data S1** Supporting Information.

**Table S1** Virus Titer of Assay Control for each Titration Assay of Clinical Specimens and Subject No. Measured in each Assay.
**Table S2** Likelihood Ratio Test for Model Selection.
**Table S3** Mixed Effect Model Based Analysis of Virus titer (log_10_TCID_50_/mL) by Day (Sensitivity Analysis).

## Data Availability

The data that support the findings of this study are available from the corresponding author upon reasonable request.
