# Supplementary figures and images for: Stability of Respiratory Syncytial Virus in Nasal Aspirate From Patients Infected With RSV
Source: Influenza Other Respir Viruses. 2024 Dec 16;18(12):e70058. doi: 10.1111/irv.70058 (PMC11649581; doi:10.1111/irv.70058)

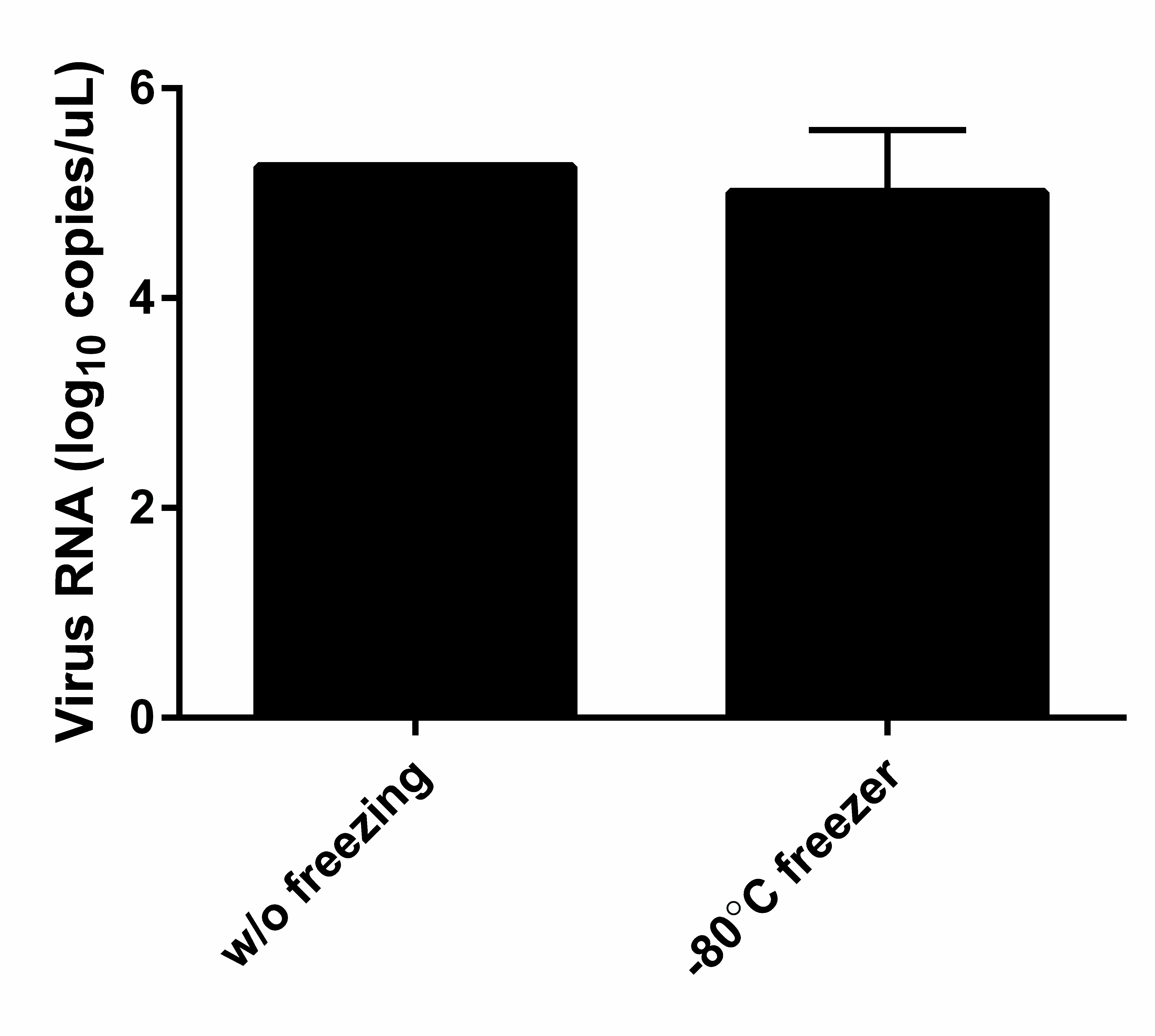

Supplement: Supplementary file 1 — Figure S1 RNA of RSV‐A2 Laboratory Strain added to VTM and Frozen in a − 80 °C freezer. One‐hundred‐fold dilution of virus stock was added to VTM and frozen in a − 80 °C freezer (n = 4). Virus RNA was then measured and is expressed as the mean ± SD. Baseline:virus RNA of samples prepared just before RNA measurement [without (w/o) freezing, n = 2; data is expressed as the mean]. [file IRV-18-e70058-s002.tif]

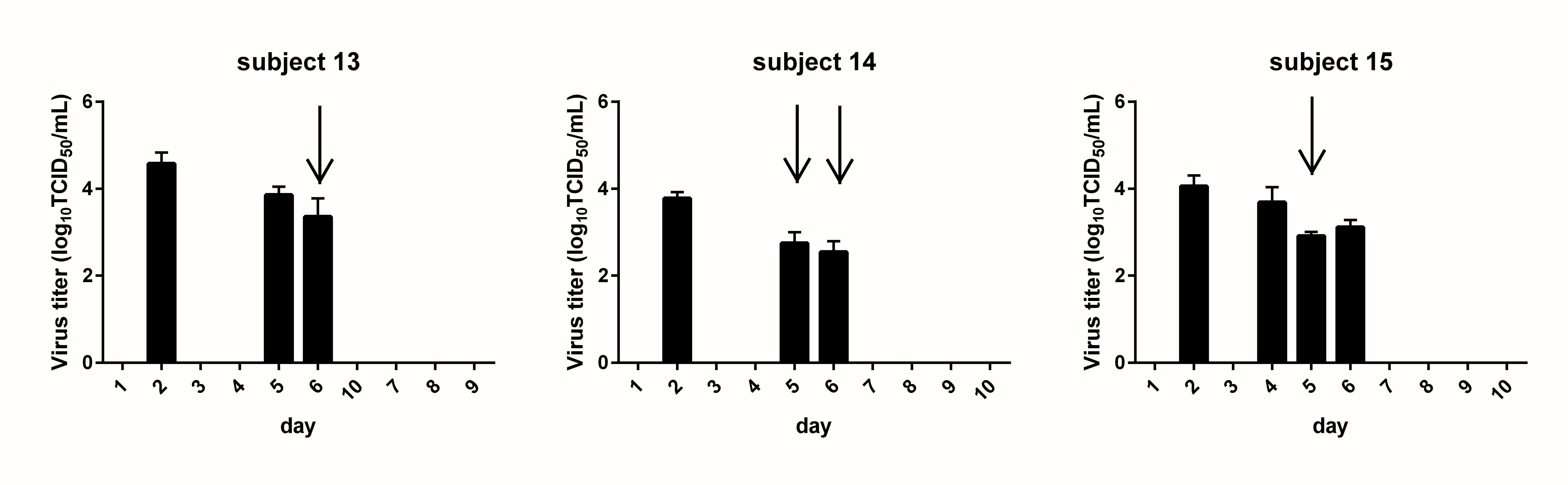

Supplement: Supplementary file 2 — Figure S2 Virus Titers of Nasal Aspirates from Subjects 13, 14 and 15 Collected into VTM and Stored at 4 °C. Three specimen aliquots were titrated at each time point and data are represented as the mean ± SD. Baseline:virus titer at first measurement time point of each subject. Black arrow:the sample showed more than 1.0 log10TCID50/mL lower virus titer from the baseline. [file IRV-18-e70058-s004.tif]
