# Supplementary material for: Stability of Respiratory Syncytial Virus in Nasal Aspirate From Patients Infected With RSV
Source: Influenza Other Respir Viruses. 2024 Dec 16;18(12):e70058. doi: 10.1111/irv.70058 (PMC11649581; doi:10.1111/irv.70058)
Supplement: Supplementary file 3 — Data S1 Supporting Information. [file IRV-18-e70058-s003.docx]

**Supplementary Information**

**Information Regarding the Virus Titer of Nasal Aspirates from Subjects 12–15 Collected and Stored in VTM at 4°C.**

Virus titration of the clinical specimen from subject 12 was not conducted because 6 days had passed from the day of sample collection to the arrival of the specimen at our laboratory. The results of subjects 13, 14 and 15 were not included in the analysis of virus stability at 4℃ because the virus titration assays at the first measurement time point (assay No. 15 and 18) were not considered to be successful. This was because the virus titer of the assay control was lower than that in the other assays (Supplementary Table 1). Although the results of subjects 13, 14 and 15 were not included in the analysis, 3 days after the first virus titer measurement, two out of the three subjects were 1.0 log_10_TCID_50_/mL lower than at the first measurement time point (Supplementary Figure 2). These results support our conclusion based on the data from the other subjects.

**Adoption of the Regression Model.**

A model with a random effect on the viral titer (intercept) on Day 1 was considered for the assessment of the relationship between RSV titer of clinical specimens stored at 4℃ and the storage period. To assess whether the slope had a random effect, two models, one without a random effect on the slope (1) and the other with a random effect (2), was examined by a likelihood ratio test.

$$y_{i,day}=\alpha+a_{i}+\beta*day+\varepsilon_{i} (1)$$

$$y_{i,day}=\alpha+a_{i}+\left( \beta+b_{i} \right)*day+\varepsilon_{i} (2)$$

Let the variance-covariance matrices of the simultaneous distributions of $a_{i}$ and $b_{i}$ be considered models with only variance components (VC) and unstructured (UN). Based on the degrees of freedom of the models, the likelihood ratio test statistics for model (1) and model (2) UN followed a chi-square distribution with two degrees of freedom, and the likelihood ratio test statistics for model (2) VC and model (2) UN followed a chi-square distribution with one degree of freedom. The P-values of the likelihood ratio test were 0.0125 and 0.0179 (< 0.05), respectively (Supplementary Table 2); therefore, model (2) UN was adopted.

**Sensitivity Analysis of the Regression Model**

A sensitivity analysis was conducted to confirm the robustness of the estimated duration of stable viral titer. One sample was excluded from the 12 samples, and the same analysis was conducted to confirm whether the estimated duration of stable viral titer was the same as that based on 12 samples. The results of the sensitivity analysis are shown in Supplementary Table 3. In all cases, the duration of stable viral titer was estimated to be 2 days, confirming the robustness of the analysis results.
