# Supplementary material for: Stability of Respiratory Syncytial Virus in Nasal Aspirate From Patients Infected With RSV
Source: Influenza Other Respir Viruses. 2024 Dec 16;18(12):e70058. doi: 10.1111/irv.70058 (PMC11649581; doi:10.1111/irv.70058)
Supplement: Supplementary file 4 — Table S1 Virus Titer of Assay Control for each Titration Assay of Clinical Specimens and Subject No. Measured in each Assay. Table S2 Likelihood Ratio Test for Model Selection. Table S3 Mixed Effect Model Based Analysis of Virus titer (log10TCID50/mL) by Day (Sensitivity Analysis). [file IRV-18-e70058-s001.docx]

**Tables of the Supplementary information**

**Supplementary Table 1. Virus Titer of Assay Control for each Titration Assay of Clinical Specimens and Subject No. Measured in each Assay.**

| Assay No. | Virus titer (log_10_TCID_50_/mL) | Subject No. measured in each assay |
| --- | --- | --- |
| 1 | 7.6 | 1, 2, 3, 4, 5 |
| 2 | 6.8 | 1, 2, 3, 4, 5 |
| 3 | 6.0 | 1, 2, 3, 4, 5 |
| 4 | 7.9 ± 0.1 | 6, 7 |
| 5 | 7.7 ± 0.3 | 6, 7 |
| 6 | 7.1 ± 0.2 | 6, 7 |
| 7 | 7.1 ± 0.2 | 8 |
| 8 | 7.1 ± 0.2 | 8 |
| 9 | 7.7 ± 0.2 | 8 |
| 10 | 7.7 ± 0.2 | 9 |
| 11 | 7.4 ± 0.4 | 9 |
| 12 | 6.5 ± 0.5 | 9 |
| 13 | 6.3 ± 0.4 | 10, 11 |
| 14 | 7.2 ± 0.3 | 10, 11 |
| 15 | 4.6 ± 0.6 | 13, 14 |
| 16 | 7.2 ± 0.4 | 13, 14 |
| 17 | 6.7 ± 0.1 | 13, 14 |
| 18 | 4.8 ± 0.2 | 15 |
| 19 | 5.4 ± 0.4 | 15 |
| 20 | 7.1 ± 0.3 | 15 |
| 21 | 7.9 ± 0.8 | 15 |
| 22 | 8.0 ± 0.2 | 16 |
| 23 | 7.9 ± 0,6 | 16 |
| 24 | 7.7 ± 0.1 | 16 |

Aliquoted RSV stock was titrated in each virus titration assay for clinical specimens as an assay control. One aliquot was titrated in assays 1–3 and three aliquots were titrated in assays 4–24. Data in assays 4–24 are represented as the mean ± SD.

**Supplementary Table 2. Likelihood Ratio Test for Model Selection**

| Model | Degree of freedom | Log likelihood | Likelihood ratio test statistic | p-value |
| --- | --- | --- | --- | --- |
| (1) | 4 | -37.3 | 8.8 | 0.0125 |
| (2) VC | 5 | -35.7 | 5.6 | 0.0179 |
| (2) UN | 6 | -32.9 |  |  |

VC = Variance component, UN = Unstructured

**Supplementary Table 3. Mixed Effect Model Based Analysis of Virus titer (log_10_TCID_50_/mL) by Day (Sensitivity Analysis)**

| Excluded  Specimen  Number | Day | Observed Value | | Model Based Value | |
| --- | --- | --- | --- | --- | --- |
|  |  | n | Mean (Standard Deviation) | LS Mean (Standard Error) | [90% Confidence Interval] |
| 1 | 1 | 1 | 4.63 (---) | 4.43 (0.12) | [4.21, 4.65] |
|  | 2 | 3 | 4.08 (0.69) | 4.16 (0.13) | [3.93, 4.40] |
|  | 3 | 8 | 3.97 (0.40) | 3.89 (0.14) | [3.63, 4.15] |
|  | 4 | 2 | 4.05 (1.06) | 3.62 (0.16) | [3.33, 3.92] |
|  | 5 | 3 | 3.58 (0.63) | 3.35 (0.19) | [3.02, 3.69] |
|  | 6 | 3 | 2.75 (0.69) | 3.08 (0.21) | [2.70, 3.47] |
|  | 7 | 4 | 2.72 (0.80) | 2.82 (0.24) | [2.38, 3.25] |
|  | 8 | 2 | 2.47 (1.65) | 2.55 (0.27) | [2.06, 3.03] |
|  | 9 | 3 | 2.3 (1.50) | 2.28 (0.30) | [1.73, 2.82] |
|  | 10 | 2 | 1.05 (0.11) | 2.01 (0.33) | [1.41, 2.60] |
| 2 | 1 | 1 | 4.63 (---) | 4.25 (0.30) | [3.70, 4.79] |
|  | 2 | 3 | 4.08 (0.69) | 4.00 (0.27) | [3.52, 4.48] |
|  | 3 | 8 | 3.78 (0.85) | 3.75 (0.24) | [3.32, 4.18] |
|  | 4 | 2 | 4.05 (1.06) | 3.50 (0.22) | [3.10, 3.90] |
|  | 5 | 3 | 3.08 (1.25) | 3.25 (0.22) | [2.86, 3.64] |
|  | 6 | 3 | 2.75 (0.69) | 3.01 (0.22) | [2.60, 3.41] |
|  | 7 | 4 | 2.72 (0.80) | 2.76 (0.25) | [2.31, 3.20] |
|  | 8 | 2 | 2.47 (1.65) | 2.51 (0.28) | [2.01, 3.01] |
|  | 9 | 3 | 2.47 (1.53) | 2.26 (0.31) | [1.69, 2.83] |
|  | 10 | 2 | 1.05 (0.11) | 2.01 (0.35) | [1.37, 2.65] |
| 3 | 1 | 1 | 4.63 (---) | 4.19 (0.30) | [3.64, 4.73] |
|  | 2 | 3 | 4.08 (0.69) | 3.93 (0.26) | [3.45, 4.40] |
|  | 3 | 8 | 3.65 (0.84) | 3.67 (0.23) | [3.25, 4.09] |
|  | 4 | 2 | 4.05 (1.06) | 3.41 (0.21) | [3.03, 3.78] |
|  | 5 | 3 | 2.74 (0.82) | 3.15 (0.19) | [2.80, 3.50] |
|  | 6 | 3 | 2.75 (0.69) | 2.89 (0.20) | [2.53, 3.24] |
|  | 7 | 4 | 2.72 (0.80) | 2.63 (0.21) | [2.25, 3.01] |
|  | 8 | 2 | 2.47 (1.65) | 2.37 (0.24) | [1.94, 2.80] |
|  | 9 | 3 | 1.97 (1.04) | 2.11 (0.27) | [1.62, 2.60] |
|  | 10 | 2 | 1.05 (0.11) | 1.85 (0.31) | [1.29, 2.41] |
| 4 | 1 | 1 | 4.63 (---) | 4.14 (0.29) | [3.61, 4.67] |
|  | 2 | 2 | 3.8 (0.71) | 3.89 (0.26) | [3.43, 4.35] |
|  | 3 | 9 | 3.73 (0.81) | 3.64 (0.22) | [3.24, 4.04] |
|  | 4 | 1 | 3.3 (---) | 3.39 (0.20) | [3.03, 3.76] |
|  | 5 | 4 | 3.13 (1.03) | 3.14 (0.19) | [2.80, 3.49] |
|  | 6 | 3 | 2.75 (0.69) | 2.89 (0.20) | [2.54, 3.25] |
|  | 7 | 4 | 2.72 (0.80) | 2.64 (0.22) | [2.25, 3.04] |
|  | 8 | 1 | 1.3 (---) | 2.40 (0.25) | [1.95, 2.85] |
|  | 9 | 4 | 2.43 (1.25) | 2.15 (0.29) | [1.63, 2.67] |
|  | 10 | 2 | 1.05 (0.11) | 1.90 (0.33) | [1.30, 2.49] |
| 5 | 1 | 0 | --- | 4.18 (0.30) | [3.63, 4.73] |
|  | 2 | 3 | 4.08 (0.69) | 3.92 (0.27) | [3.44, 4.40] |
|  | 3 | 8 | 3.65 (0.84) | 3.67 (0.23) | [3.25, 4.10] |
|  | 4 | 2 | 4.05 (1.06) | 3.42 (0.21) | [3.04, 3.80] |
|  | 5 | 4 | 3.13 (1.03) | 3.17 (0.20) | [2.80, 3.53] |
|  | 6 | 3 | 2.75 (0.69) | 2.92 (0.21) | [2.54, 3.29] |
|  | 7 | 3 | 2.36 (0.42) | 2.66 (0.23) | [2.25, 3.07] |
|  | 8 | 2 | 2.47 (1.65) | 2.41 (0.25) | [1.95, 2.87] |
|  | 9 | 4 | 2.43 (1.25) | 2.16 (0.29) | [1.63, 2.69] |
|  | 10 | 2 | 1.05 (0.11) | 1.91 (0.33) | [1.31, 2.51] |
| 6 | 1 | 1 | 4.63 (---) | 4.19 (0.30) | [3.64, 4.73] |
|  | 2 | 3 | 4.08 (0.69) | 3.96 (0.27) | [3.48, 4.44] |
|  | 3 | 8 | 3.74 (0.87) | 3.73 (0.24) | [3.30, 4.17] |
|  | 4 | 2 | 4.05 (1.06) | 3.51 (0.22) | [3.11, 3.90] |
|  | 5 | 4 | 3.13 (1.03) | 3.28 (0.21) | [2.90, 3.67] |
|  | 6 | 3 | 2.75 (0.69) | 3.05 (0.22) | [2.66, 3.45] |
|  | 7 | 3 | 2.97 (0.76) | 2.83 (0.24) | [2.40, 3.25] |
|  | 8 | 2 | 2.47 (1.65) | 2.60 (0.26) | [2.12, 3.08] |
|  | 9 | 4 | 2.43 (1.25) | 2.37 (0.30) | [1.83, 2.91] |
|  | 10 | 1 | 1.13 (---) | 2.14 (0.34) | [1.53, 2.76] |
| 7 | 1 | 1 | 4.63 (---) | 4.25 (0.30) | [3.71, 4.80] |
|  | 2 | 2 | 4.47 (0.23) | 4.02 (0.26) | [3.55, 4.50] |
|  | 3 | 9 | 3.73 (0.81) | 3.79 (0.23) | [3.38, 4.21] |
|  | 4 | 2 | 4.05 (1.06) | 3.56 (0.21) | [3.19, 3.93] |
|  | 5 | 4 | 3.13 (1.03) | 3.33 (0.20) | [2.98, 3.68] |
|  | 6 | 2 | 3.14 (0.23) | 3.10 (0.20) | [2.74, 3.46] |
|  | 7 | 4 | 2.72 (0.80) | 2.87 (0.22) | [2.48, 3.26] |
|  | 8 | 2 | 2.47 (1.65) | 2.64 (0.24) | [2.19, 3.08] |
|  | 9 | 3 | 2.97 (0.76) | 2.41 (0.28) | [1.90, 2.92] |
|  | 10 | 2 | 1.05 (0.11) | 2.18 (0.32) | [1.59, 2.76] |
| 8 | 1 | 1 | 4.63 (---) | 4.18 (0.30) | [3.63, 4.72] |
|  | 2 | 3 | 4.08 (0.69) | 3.95 (0.27) | [3.47, 4.43] |
|  | 3 | 8 | 3.72 (0.87) | 3.73 (0.24) | [3.29, 4.16] |
|  | 4 | 2 | 4.05 (1.06) | 3.50 (0.22) | [3.10, 3.90] |
|  | 5 | 4 | 3.13 (1.03) | 3.28 (0.21) | [2.89, 3.66] |
|  | 6 | 3 | 2.75 (0.69) | 3.05 (0.22) | [2.65, 3.45] |
|  | 7 | 4 | 2.72 (0.80) | 2.82 (0.24) | [2.40, 3.25] |
|  | 8 | 1 | 3.63 (---) | 2.60 (0.26) | [2.12, 3.07] |
|  | 9 | 4 | 2.43 (1.25) | 2.37 (0.29) | [1.84, 2.91] |
|  | 10 | 1 | 0.97 (---) | 2.15 (0.33) | [1.54, 2.75] |
| 9 | 1 | 1 | 4.63 (---) | 4.17 (0.30) | [3.63, 4.71] |
|  | 2 | 3 | 4.08 (0.69) | 3.94 (0.26) | [3.46, 4.41] |
|  | 3 | 8 | 3.68 (0.85) | 3.70 (0.24) | [3.27, 4.13] |
|  | 4 | 2 | 4.05 (1.06) | 3.46 (0.22) | [3.06, 3.87] |
|  | 5 | 3 | 3.13 (1.26) | 3.23 (0.22) | [2.84, 3.62] |
|  | 6 | 3 | 2.75 (0.69) | 2.99 (0.22) | [2.59, 3.40] |
|  | 7 | 3 | 2.69 (0.98) | 2.76 (0.24) | [2.32, 3.20] |
|  | 8 | 2 | 2.47 (1.65) | 2.52 (0.27) | [2.03, 3.02] |
|  | 9 | 4 | 2.43 (1.25) | 2.29 (0.31) | [1.73, 2.85] |
|  | 10 | 2 | 1.05 (0.11) | 2.05 (0.35) | [1.42, 2.69] |
| 10 | 1 | 1 | 4.63 (---) | 4.18 (0.30) | [3.64, 4.73] |
|  | 2 | 3 | 4.08 (0.69) | 3.95 (0.27) | [3.47, 4.43] |
|  | 3 | 8 | 3.72 (0.87) | 3.72 (0.24) | [3.29, 4.15] |
|  | 4 | 2 | 4.05 (1.06) | 3.49 (0.22) | [3.09, 3.89] |
|  | 5 | 4 | 3.13 (1.03) | 3.26 (0.22) | [2.87, 3.65] |
|  | 6 | 3 | 2.75 (0.69) | 3.03 (0.22) | [2.62, 3.43] |
|  | 7 | 3 | 2.86 (0.92) | 2.79 (0.24) | [2.36, 3.23] |
|  | 8 | 2 | 2.47 (1.65) | 2.56 (0.27) | [2.07, 3.05] |
|  | 9 | 4 | 2.43 (1.25) | 2.33 (0.30) | [1.78, 2.88] |
|  | 10 | 2 | 1.05 (0.11) | 2.10 (0.34) | [1.48, 2.72] |
| 11 | 1 | 1 | 4.63 (---) | 4.18 (0.30) | [3.63, 4.72] |
|  | 2 | 2 | 3.97 (0.94) | 3.94 (0.27) | [3.46, 4.42] |
|  | 3 | 9 | 3.73 (0.81) | 3.70 (0.24) | [3.26, 4.13] |
|  | 4 | 2 | 4.05 (1.06) | 3.45 (0.22) | [3.06, 3.85] |
|  | 5 | 4 | 3.13 (1.03) | 3.21 (0.21) | [2.83, 3.60] |
|  | 6 | 2 | 2.47 (0.71) | 2.97 (0.22) | [2.57, 3.37] |
|  | 7 | 4 | 2.72 (0.80) | 2.73 (0.24) | [2.29, 3.17] |
|  | 8 | 2 | 2.47 (1.65) | 2.49 (0.27) | [2.00, 2.98] |
|  | 9 | 4 | 2.43 (1.25) | 2.25 (0.31) | [1.69, 2.80] |
|  | 10 | 2 | 1.05 (0.11) | 2.01 (0.35) | [1.38, 2.63] |
| 16 | 1 | 1 | 4.63 (---) | 4.14 (0.29) | [3.61, 4.68] |
|  | 2 | 3 | 4.08 (0.69) | 3.92 (0.26) | [3.44, 4.39] |
|  | 3 | 8 | 3.63 (0.82) | 3.69 (0.24) | [3.26, 4.12] |
|  | 4 | 1 | 4.8 (---) | 3.46 (0.22) | [3.06, 3.86] |
|  | 5 | 4 | 3.13 (1.03) | 3.23 (0.22) | [2.84, 3.62] |
|  | 6 | 2 | 2.64 (0.94) | 3.00 (0.22) | [2.60, 3.41] |
|  | 7 | 4 | 2.72 (0.80) | 2.77 (0.24) | [2.34, 3.21] |
|  | 8 | 2 | 2.47 (1.65) | 2.55 (0.27) | [2.06, 3.03] |
|  | 9 | 4 | 2.43 (1.25) | 2.32 (0.30) | [1.77, 2.86] |
|  | 10 | 2 | 1.05 (0.11) | 2.09 (0.34) | [1.48, 2.70] |
